# Impact of Environmental Conditions and Agronomic Practices on the Prevalence of *Fusarium* Species Associated with Ear- and Stalk Rot in Maize

**DOI:** 10.3390/pathogens9030236

**Published:** 2020-03-21

**Authors:** Annette Pfordt, Lucia Ramos Romero, Simon Schiwek, Petr Karlovsky, Andreas von Tiedemann

**Affiliations:** 1 Plant Pathology and Crop Protection, University of Goettingen, 37077 Goettingen, Germany; 2 Molecular Phytopathology and Mycotoxin Research, University of Goettingen, 37077 Goettingen, Germany

**Keywords:** *Fusarium* spp., ear rot, stalk rot, maize, monitoring, weather conditions, agronomic practice

## Abstract

*Fusarium* species are common pathogens on maize and reduce the product quality through contamination with mycotoxins thus jeopardizing safety of both animal feed and human food products. Monitoring of *Fusarium* infected maize ears and stalks was conducted in Germany to determine the range of *Fusarium* species present in the field and to assess the impact of tillage, crop rotation, and weather conditions on the frequency of *Fusarium* species. From 2016 till 2018, a total of 387 infected ears and 190 stalk segments from 58 locations in Germany were collected. For each sample location, site-specific agronomic data on tillage and previous crops as well as meteorological data on precipitation, air temperature, and relative humidity during the vegetation period were recorded. The most frequent *Fusarium* species detected in maize ears were *Fusarium* *graminearum*, *F.* *verticillioides* and *F.* *temperatum*, whereas, *F.* *graminearum*, *F.* *equiseti*, *F.* *culmorum*, and *F.* *temperatum* were the species prevailing on maize stalks. Differences in the local species composition were found to be primarily associated with weather variations between the years and the microclimate at the different locations. The results indicate that mean temperature and precipitation in July, during flowering, has the strongest impact on the local range of *Fusarium* spp. on ears, whereas the incidence of *Fusarium* species on stalks is mostly affected by weather conditions during September. Ploughing significantly reduced the infection with *F.* *graminearum* and *F.* *temperatum*, while crop rotation exerted only minor effects.

## 1. Introduction

*Fusarium* spp. in maize occur worldwide and can cause various diseases in different growth stages of maize, such as root and seedling rot as well as stalk and ear rot [1]. *Fusarium* ear rot (FER) and *Fusarium* stalk rot (FSR) are characterized by a white or reddish discoloration with rotting symptoms on the ears and inside the stalk [2,3]. Several toxigenic *Fusarium* species are known to cause yield losses, reduction of grain quality, thus endangering the safety of both animal feed and human food products [4,5]. The dominant *Fusarium* species causing ear and stalk rot in temperate climate zones are *Fusarium graminearum*, *F. verticillioides*, and *F. subglutinans*, producing numerous, chemically diverse mycotoxins, among which the most important are deoxynivalenol, zearalenon, and fumonisin [6,7].

Previous studies demonstrated, that the local *Fusarium* species composition is influenced by weather conditions as well as cultural practices [8,9,10,11,12,13]. Several routes are known by which the fungus may enter the kernels and the stalk including wounds by insects [14,15], silk infection and systemic spread after root penetration [1,16]. The major infection pathway for the infection of maize ears by most *Fusarium* species is via the silk channel with highest severity occurring at early stages of silk development [17,18]. In contrast, infection with *F. verticillioides* is often associated with injury by insects, primarily due to the feeding of the European corn borer (*Ostrinia nubilalis*), at 10–15 days after silk emergence. Stalk colonization has been reported to increase late in the season [19,20] due to an increase in tissue susceptibility when carbohydrates and other nutrients are redirected towards developing kernels [21]. The importance of infection pathways and timepoints of infection may vary among geographical regions due to differences in weather conditions and the occurrence of insects. Temperature and moisture appear to be the most important factors affecting the range of *Fusarium* species of ear and stalk rot infection. Favorable weather conditions for an infection of *Gibberella* ear rot, mainly caused by *F. graminearum* and *F. culmorum* are low temperatures and high precipitation, whereas infection with *F. verticillioides*, *F. subglutinans*, and *F. proliferatum* (*Fusarium* ear rot) is promoted at high temperatures and dry conditions [22,23].

Likewise, cultural practices such as crop rotation and tillage have been reported to influence the disease incidence and severity of *Fusarium* infection in wheat and maize [24]. Residues of previous crops serve as source of inoculum for subsequent infection [25,26] and also promote the survival of *Ostrinia nubilalis*, which may further enhance the risk of infection with *Fusarium* spp. [1,16]. Controversial effects of tillage and crop residues have been reported in previous studies. Some reports indicated no effect of alternating corn tillage practices on the incidence of ear rot pathogens [10,27,28,29], whereas others found a significant decrease in the diversity of *Fusarium* spp. in soil after conventional ploughing as compared to reduced tillage [9,30,31].

Prevention of *Fusarium* infection focuses on cultural practices such as crop rotation and ploughing as well as improving host resistance. The success of these strategies has, however, been limited owing to the broad range of *Fusarium* species and large variation in host species and their genotypes. In addition, maize growing areas with short rotations of wheat and maize increased in recent years resulting in a higher risk of *Fusarium* ear and stalk infection and mycotoxin contamination [23,32]. The complex of *Fusarium* species may also have extended and shifted due to climate variations and more intense maize cultivation [33].

Therefore, the objective of this study was to determine the actual *Fusarium* species composition of maize fields in Germany and to estimate how the frequency of local *Fusarium* species is affected by cultural practices (tillage and previous crop) and weather conditions (air temperature and precipitation) under natural infection from 2016 to 2018.

## 2. Results

### 2.1. Fusarium Species Involved in Ear and Stalk Infections

In the three years of field investigations, a total number of 11,610 kernels and 3483 rachis and stalk samples were analyzed to determine the local *Fusarium* spp. composition. In the years 2017 and 2018, twelve *Fusarium* species were identified. In 2016, *F. verticillioides* and *F. proliferatum* as well as *F. temperatum* and *F. subglutinans* were treated as a species complex of *F. proliferatum sp.* and *F. subglutinans sp*., respectively (Table 1).

In 2016 and 2017, *F. graminearum* was the predominant species in maize ears and detected in over 60% of all tested samples. The detection frequency of *F. graminearum* differed from year to year, with 79% in 2016, 71% in 2017 and 30% in 2018. *F. verticillioides* was the prevailing species in 2018 and detected in 39% of all ears. In total, *F. verticillioides* colonized 24% of all tested ears from 38 locations. Detection frequency of *F. temperatum* ranged from 15% in 2017 up to 33% in 2016. In total, 23% of all ears analyzed were infected with *F. temperatum*. *F. poae* colonized 14% of all tested ears followed by minor species such as *F. cerealis* (9%), *F. proliferatum* (6%), *F. tricinctum* (5%), *F. avenaceum* (5%), *F. culmorum* (4%), *F. subglutinans* (2%), *F. equiseti* (2%), and *F. sporotrichioides* (2%). Similar to ears, *F. graminearum* was also prevailing on maize stalks where; it was detected in 62% of all tested samples. In 2017, *F. graminearum* was present in more than 80% of the stalks and occurred at almost each sampling location. *F. equiseti* colonized a total of 22% of the samples within two years of investigation, however, the percentage of infected stalks containing *F. equiseti* was much higher in 2018 (34%) compared to 2017 (11%). *F. culmorum* was the third most frequent species isolated from 22% of the stalks in 2017 and 16% in 2018. Infection with *F. temperatum* and *F. cerealis* was found in 17% of the stalk samples, however, *F. cerealis* was more frequent in 2017 (19%) and *F. temperatum* in 2018 (20%). *F. verticillioides*, *F. avenaceum*, *F. tricinctum*, *F. poae F. subglutinans*, and *F. sporotrichioides* were detected less frequently.

### 2.2. Effect of Previous Crop

**Figure 1:**
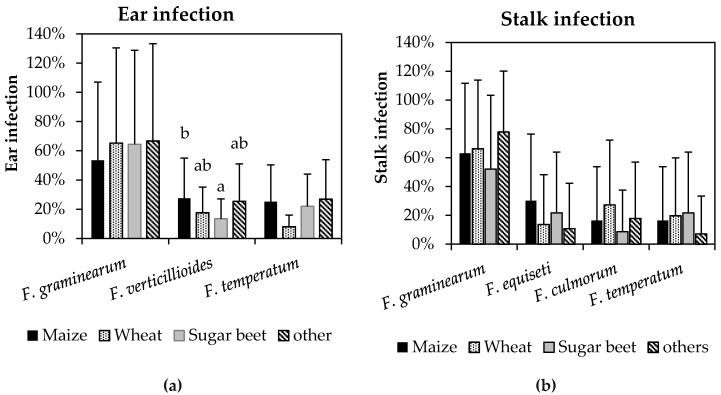
Percentage of ears (**a**) infected with *F. graminearum, F. verticillioides,* and *F. temperatum* and stalks (**b**) infected with *F. graminearum, F. equiseti, F. culmorum,* and *F. temperatum* depending on the previous crop (maize, wheat, sugar beet, others). Vertical bars represent standard deviations. Different letters indicate significant differences (*p* ≤ 0.05) within species.

The abundance of the three most frequent species on ears (*F. graminearum*, *F. verticillioides*, and *F. temperatum*) and the four species prevailing on stalks (*F. graminearum*, *F. equiseti*, *F. culmorum*, and *F. temperatum*) after different pre-crops is shown in Figure 1. Crop rotation had no significant effect on ear and stalk infection with *F. graminearum*, *F. temperatum*, *F. equiseti*, and *F. culmorum*. The frequency of *F. graminearum* on ears was slightly reduced in maize after maize as compared to wheat, sugar beet and non-host crops like potato, strawberries or cabbage. The highest frequency of stalk infection with *F. graminearum* was observed on maize after other crops, followed by wheat, maize and sugar beet. No effects of pre-crops were found for ears infected with *F. temperatum.* Maize as previous crop slightly favored stalk infection with *F. equiseti* (30%) while colonization with *F. culmorum* was slightly increased after wheat (27%). Only *F. verticillioides* indicated strong differences in frequency of ear infection. Colonization of *F. verticillioides* was significantly favored by maize after maize in comparison to maize after sugar beet.

### 2.3. Effect of Tillage

**Figure 2:**
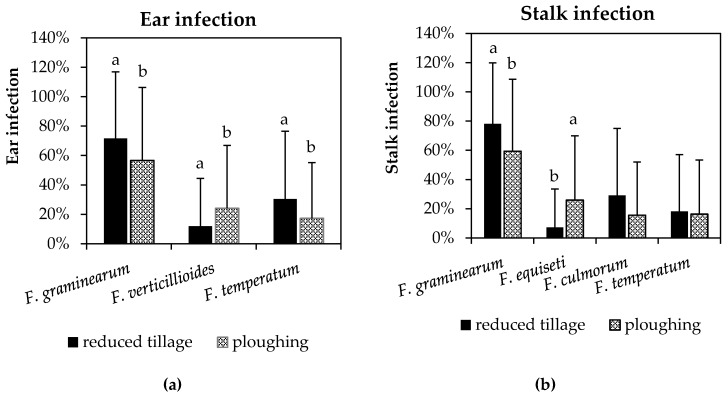
Percentage of ears (**a**) infected with *F. graminearum*, *F. verticillioides*, and *F. temperatum* and stalk (**b**) infected with *F. graminearum*, *F. equiseti*, *F. culmorum*, and *F. temperatum* depending on tillage (reduced tillage vs. ploughing). Vertical bars represent standard deviation. Different letters indicate significant differences (*p* ≤ 0.05) within species.

Ear and stalk infection with *F. graminearum*, *F. verticillioides*, and *F. equiseti* were significantly affected by the type of soil tillage as shown in Figure 2. Apart from *F. verticillioides* and *F. equiseti*, reduced tillage (chisel ploughing and rotary harrow) favored infection with most *Fusarium* species compared to moldboard ploughing. Hence, colonization with *F. graminearum* in ear and stalk samples was significantly higher at reduced tillage compared to moldboard ploughing. Similarly, ear infection with *F. temperatum* was reduced after ploughing (30%) compared to reduced tillage (17%). Ploughing also reduced the frequency of *Fusarium* species in maize stalks, however, it led to higher frequencies in observations with *F. equiseti*. The percentage of ears colonized with *F. verticillioides* was significantly higher after ploughing (24%) than after reduced tillage (12%). *F. equiseti* was equally favored by ploughing (28%) as compared to reduced tillage (7%). The type of tillage had no significant effect on stalk infection with *F. culmorum* and *F. temperatum*.

### 2.4. Effect of Environmental Conditions

**Figure 3:**
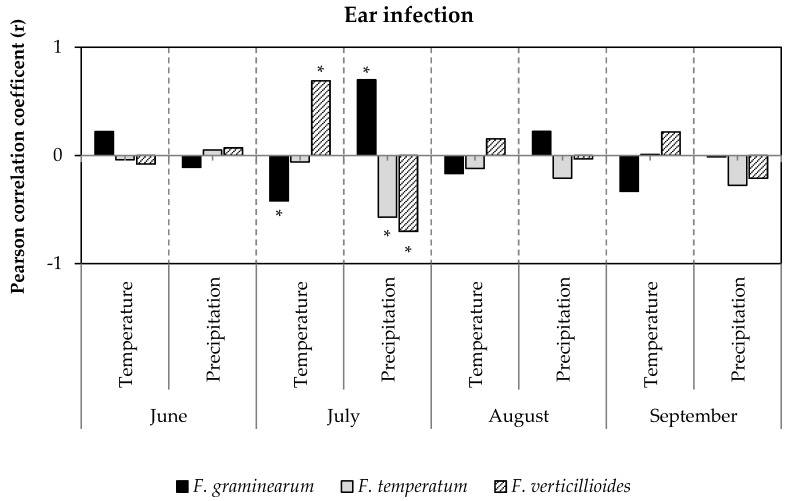
Coefficients of correlation of temperature and precipitation during June, July, August, and September with ear infection by *F. graminearum*, *F. temperatum* and *F. verticillioides*. Bars represent coefficients of correlation between percentage of sampled ears per location infected with *F. graminearum*, *F. temperatum*, and *F. verticillioides* and weather data at the sampling sites recorded in 2016, 2017 and 2018 (n = 387). Asterisk (*) indicates statistically significant correlation (*p* ≤ 0.05).

**Figure 4:**
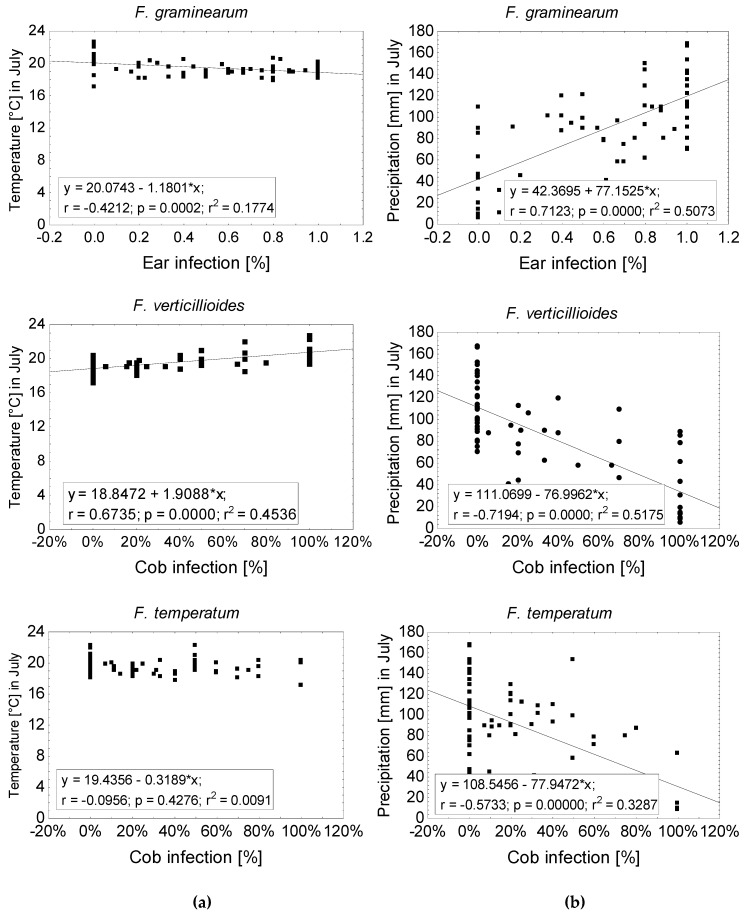
Relationship between ear infection [%] and temperature (**a**) or precipitation (**b**) in July of *F. graminearum*, *F. equiseti*, *F. culmorum*, and *F. temperatum*. Solid line indicates a statistically significant (*p* ≤ 0.05) least squares linear relationship.

The relationship between the frequency of *Fusarium* species on ears (*F. graminearum*, *F. verticillioides*, and *F. temperatum*) and stalks (*F. graminearum*, *F. equiseti*, *F. culmorum*, and *F. temperatum*) and weather conditions after flowering was analyzed using Pearson correlation (Supplementary Table S1). While temperature and precipitation in June had no significant effect on the occurrence of the most frequent *Fusarium* species (Figure 3), temperature and precipitation during flowering in July had a medium to strong effect on the frequency of the prevailing species. Colonization of *F. graminearum* negatively correlated (r = −0.42) with temperature in July and positively correlated (r = 0.70) with precipitation in July. *F. temperatum* was favored by low precipitation (r = −0.71) and *F. verticillioides* was found to be more frequent at high temperatures (r = 0.69) and low precipitation (r = −0.71). Temperature and precipitation during August and September had minor effects on frequencies of *Fusarium* species. The correlations described above demonstrate the critical impact of temperature and precipitation in July on ear infection with the most frequent *Fusarium* species (Figure 4). Frequency of *F. graminearum* was inversely related to temperature (r = −0.42) and positively correlated with precipitation in July (r = −0.71). Ear infection with *F. verticillioides* significantly increased with temperature (r = 0.67) and low precipitation (r = 0.72). The temperature in July had no effect on colonization with *F. temperatum*, however, dry conditions promoted (r^2^ = −0.57) infections of the ear.

**Figure 5:**
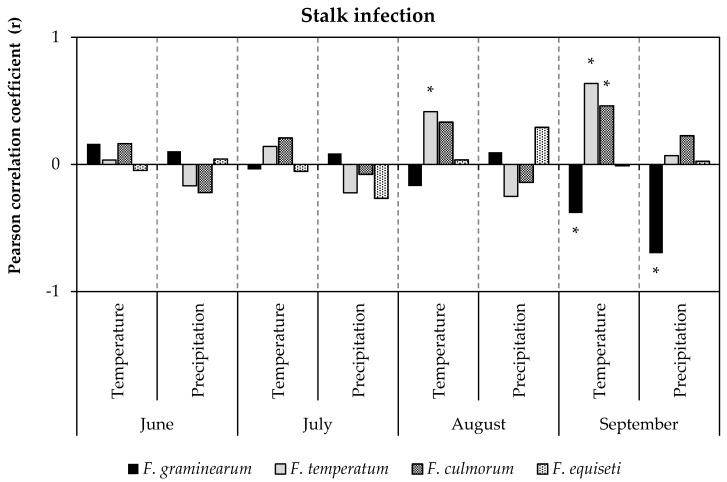
Coefficients of correlation of temperature and precipitation during June, July, August, and September with stalk infection by *F. graminearum*, *F. temperatum*, *F. culmorum*, and *F. equiseti*. Bars represent coefficients of correlation between percentage of stalks sampled per location infected with *F. graminearum*, *F. temperatum*, *F. culmorum*, and *F. equiseti* and weather data recorded at the sampling sites in 2017 and 2018 (n = 190). Asterisk (*) indicates statistically significant (*p* ≤ 0.05) correlation.

**Figure 6:**
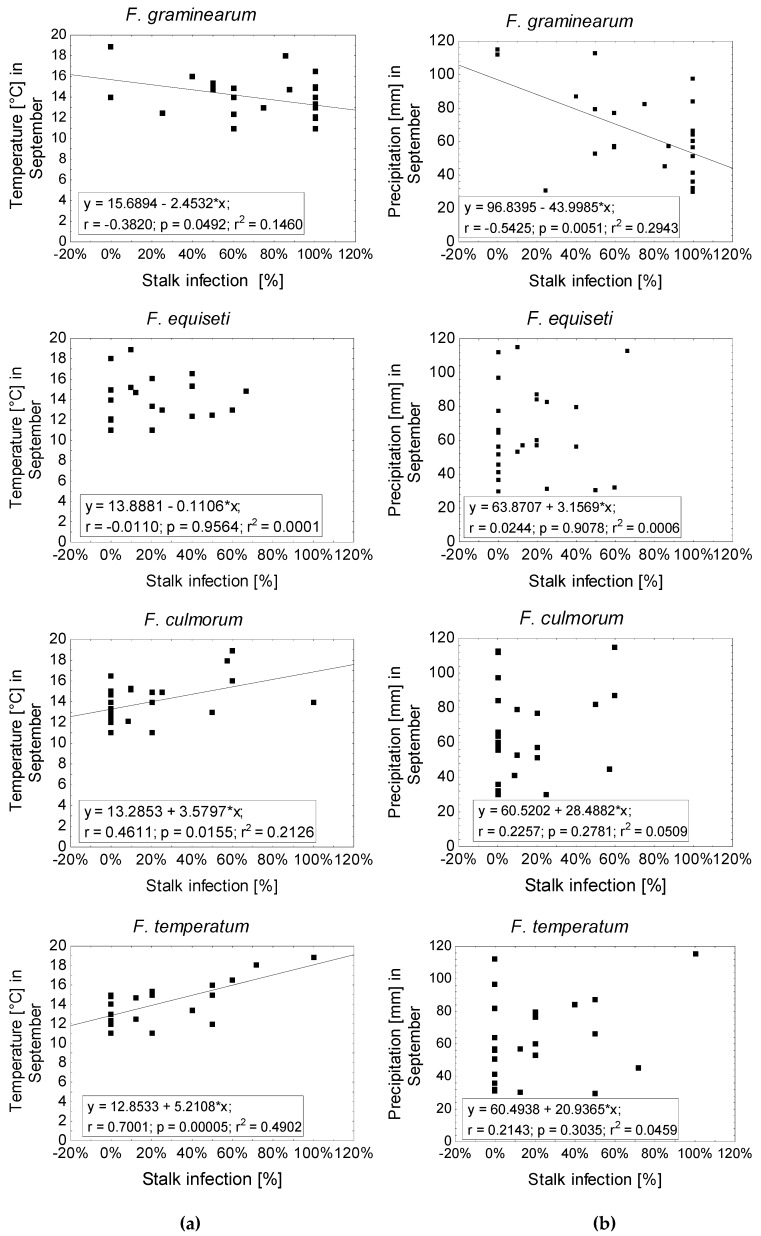
Relationship between stalk infection [%] with *F. graminearum*, *F. equiseti*, *F. culmorum*, and *F. temperatum* and temperature (**a**) or precipitation (**b**) in September. Solid lines indicate a statistically significant (*p* ≤ 0.05) least squares linear relationship.

In contrast to ear infection, the abundance of the most frequent *Fusarium* species on stalks (*F. graminearum*, *F. equiseti*, *F. culmorum*, and *F. temperatum*) displayed significant correlation with temperature and precipitation during the month of September (Figure 5). *F. graminearum* was significantly enhanced at low temperature (r = 0.38) and low precipitation (r = −0.54). However, *F. temperatum* (r = 0.63) and *F. culmorum* (r = 0.46) were favored by high temperature. Temperature and precipitation had no effect on the frequency of stalk infection with *F. equiseti*. The specific relationship between temperature and precipitation in September on one hand and stalk infection with the most frequent *Fusarium* species on the other hand (Figure 6) revealed increased frequency of *F. graminearum* at low temperatures (r = −0.38) and dry conditions (r = −0.54). In turn, the percentage of ears infected with *F. temperatum* (r = 0.70) and *F. culmorum* (r = 0.46) increased at higher temperatures. Precipitation in September had no effect on stalk infection neither with *F. temperatum* nor *F. culmorum*. Stalk infection with *F. equiseti* was not influenced by temperature or precipitation during ripening.

### 2.5. Relative Impact of Main Effects

**Figure 7:**
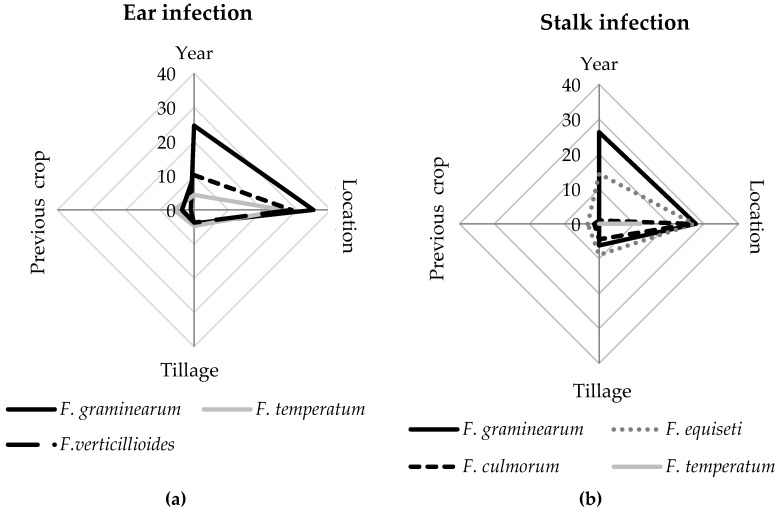
Relative impact of main effects (year, location, tillage, previous crop) expressed as percentage of variance of the total variance estimated with the restricted maximum likelihood model. Ear infection (**a**) calculated for *F. graminearum*, *F. temperatum*, and *F. verticillioides*. Stalk infection (**b**) calculated for *F. graminearum*, *F. equiseti*, *F. culmorum*, and *F. temperatum*.

The effects of tillage, previous crop, year and location on the percentage of ears and stalks infected with the most frequent *Fusarium* species were compared using the variance components of each factor (Figure 7). The strongest effect was found for the sampling location, which affected the infection of maize ears with *F. graminearum* (34.9% of variance), *F. verticillioides* (26.3%), and *F. temperatum* (28.5%). Furthermore, the frequency of *F. graminearum* was influenced by the year of sampling (24%), less by the type of tillage (3.9%) and the previous crop (1%). Similarly, the occurrence of *F. verticillioides* and *F. temperatum* were most strongly determined by the location, followed by year, previous crop and tillage. Tillage and previous crop had minor effects on ear colonization with any of the tested *Fusarium* species.

Stalk infection with *F. graminearum*, *F. equiseti*, *F. culmorum*, and *F. temperatum* was mainly affected by the year and location. Stalk infection with *F. graminearum* (26.3%) strongly differed between the years, while stalk infection with *F. equiseti* (27.1%)*, F. culmorum* (26.4%), and *F. temperatum* (11.8%) was mainly affected by the location. The previous crop had almost no effect on *Fusarium* species composition and tillage only slightly influenced the colonization with *F. graminearum* (6.2%), *F. equiseti* (8.9%), and *F. culmorum* (4.4%).

## 3. Discussion

Within the three years of investigations of maize ears and stalks, twelve *Fusarium* species were isolated and identified. All the species detected are known to frequently occur on maize ears and stalks in Central Europe [34]. High year to year variability was observed for the frequency of *Fusarium* spp., which indicated a major impact of temperature and precipitation during the vegetation period. The growing seasons in 2016 and 2017 were characterized by moderate temperatures (18.8 °C) and high precipitation in July (110 mm), while in 2018 high mean temperatures (20.6 °C) and dry conditions (40 mm in July) prevailed. This might explain the high frequency of *F. graminearum* and *F. culmorum* in 2016 and 2017 when more than 70% of all tested ears and 80% of all tested stalks were colonized with these two species. In contrast, *F. verticillioides* was the prevailing species in 2018, colonizing almost 40% of all ears analyzed.

Numerous studies confirm that moderate temperature and high level of moisture increases infection rates of *F. graminearum*, *F. culmorum*, and *F. avenaceum* (*Gibberella* ear rot) [22,25,35], while Pink ear rot pathogens such as *F. verticillioides*, *F. proliferatum*, and *F. temperatum* have often been reported from southern European regions where dry and warm conditions prevail [36]. These patterns correspond to different temperature optima of the species. The optimal growth rate for *F. graminearum* was reported 24–26 °C [37] and high level of moisture, whereas optimal conditions of *F. verticillioides* are 30 °C and 0.97 water activity [2,38]. The primary infection pathway for ear infection is via the silk channel (during the first 6 to 10 days after silk emergence) or insect injury of kernels (during grain filling). Under mid European conditions, silk emergence takes place between beginning of July and mid-July. At this time, weather conditions as well as insect populations strongly affect *Fusarium* spp. infection [1,16]. *Gibberella* ear rot pathogens are favored by high levels of moisture during silking, followed by moderate temperatures and high precipitation during cob maturation [25]. Shelby et al. [39] demonstrated that fumonisin levels and the occurrence of *Fusarium* ear rot pathogens were inversely correlated with rainfall in June and July. In particular, drought stress is associated with an elevated infection with *F. verticillioides* [40]. The present study confirmed that a dry period with high temperatures before and during grain filling favors ear infection with *F. verticillioides* and *F. temperatum*, while the frequency of *F. graminearum* was higher at lower temperatures and high precipitation. While temperature, precipitation and relative humidity during flowering were incorporated into forecasting models for *Fusarium* head blight on wheat [41], available risk assessment models for *Fusarium* ear rot disease were not sufficiently detailed to maize and cannot be extrapolated from the existing risk assessment models for *Fusarium* head blight [42]. Only Stewart et al. [43] were able to develop a mechanistic model relating the growth rates of *F. graminearum* and *F. verticillioides* to temperature, relative humidity, and precipitation which effectively predicted ear rot severity after artificial inoculation.

In contrast to ear infection, stalk infection was mainly influenced by temperature during ripening in August and September. *Fusarium* species can enter the stalk during the whole vegetation period by systemic spread after colonization of the root [1,44], through young leaf sheaths, by seed transmission [45] and via wounds caused by hail or feeding of insects [3]. Consequently, stalk rot infection is not restricted to a specific time point and fungal infection does not correlate with seasonal weather conditions. However, temperature substantially affected the extent of invasion of *Fusarium* pathogens during ripening [30,44]. Murillo-Williams and Munkvold [44] suggested that higher temperatures in particular lead to faster maturity of the plants promoting systemic infections of species which are adapted to warmer temperature such as *F. verticillioides*. Stalk rot usually occurs at physiological maturity, in August and September, when storage products in stalks are depleted and most carbohydrates are translocated to the cob [46]. Accordingly, Dodd [21] reported that at maturity stages the root and lower stalk tissues lose their metabolic activity and thus their defense potential against stalk infection. In addition, further stresses such as drought, high plant density, leaf diseases, and corn borer attacks may also favor stalk rot due to decreasing photosynthesis rate [21]. *F. temperatum*, a species recently separated from *F. subglutinans* based on its phylogeny and mycotoxin production, colonized up to 20% of all analyzed ear and stalk samples. The frequency of *F. subglutinans* within the three years of investigation was low (2%); *F. subglutinans* therefore played only a minor role in ear and stalk infection. A higher incidence of *F. temperatum* in comparison to *F. subglutinans* was also reported from maize in Belgium [47], Poland [48], France [49], and Italy [50] as well as North America [51], Korea, [52], China [53], Mexico [54], and Argentina [55]. The data of the present study demonstrate that kernel colonization with *F. temperatum* was significantly favored by low precipitation during flowering in 2018. Moretti et al. [56] suggested that isolates belonging to group 1 (*F. temperatum*) are more frequent in cooler regions like Germany, Poland and Austria while group 2 (*F. subglutinans*) prevails in warmer and dryer regions such as Slovakia, Italy and Serbia. Czembor et al. [48] reported a similar trend of *F. temperatum* occurring more often in environments with mean temperatures of 18 °C or lower in June, like in Germany.

In the present study, only low or no impact of crop rotation on ear and stalk infection with *Fusarium* spp. was observed. These findings correspond with the results from investigations by Dill-Macky and Jones [24] and Schaafsma et al. [57] indicating that similarly high disease levels caused by *F. graminearum* are found in maize grown after maize and wheat, compared to sugar beet and other pre-crops like rape seed, potato, or strawberries. A similar tendency was observed by Schlüter and Kropf [29] and Gödecke [58], who reported a high disease incidence by *F. culmorum* and *F. graminearum* on wheat after non-host crops like oilseed rape and sugar beet. Mansfield et al. [9] also reported no effect of crop rotation with broadleaf crops on DON contamination of maize stalks. The most important source of inoculum for *Fusarium* spp. are plant debris, especially maize stalks. However, these fungi are also pathogenic in cereals such as wheat, barley, oats and rye as well as sugar beet. *Fusarium* spp. can survive as mycelium and other structures on residues of these crops as well as on senescent tissue of other crops or weed species, which may later serve as primary inoculum for infection [59]. Resting structures such as chlamydospores and thick-walled hyphae can survive up to ten years breaks between host crops on plant residues buried at 30 cm depth or left on the soil surface [25,60]. Long-term survival studies by Cotton and Munkvold [26] indicate an equal survival of *Fusarium* species in buried residues and surface residues after 343 days and suggest that surface residues may act as a reservoir of recolonization and spore production for airborne inoculum and spread into the next vegetation period.

Therefore, management of surface residues by tillage and deep burial are suggested as an important strategy to control ear and stalk rot diseases [61]. The results of this study indicate that, the incidence of local *Fusarium* species on ears and stalks is highly affected by conventional ploughing compared to chisel ploughing or no tillage. In particular, the frequency of *F. graminearum*, *F. temperatum*, and *F. culmorum* was reduced after conventional ploughing, however, *F. verticillioides* and *F. equiseti* were enhanced by ploughing. Our study confirms the results reported by Dill-Macky and Jones [24] and Steinkellner and Langer [62], which demonstrated that most *Fusarium* species were reduced after moldboard ploughing as compared to reduced tillage. Covering crop residues with soil accelerates their decomposition by enhancing microbial activity and this reduces inoculum density [63,64]. However, investigations by Byrnes and Carroll [65] confirmed higher severity of *F. equiseti* infection after conventional tillage, whereas the concentration of DON produced by *F. graminearum* and the population density of *F. subglutinans* were reduced by conventional tillage [11,13,30]. Steinkellner and Langer [62] emphasized that ploughing compared to chisel plough and rotary tiller treatments reduced the number of colony forming units (CFU) per g of soil and the frequency of *Fusarium* species in upper soil layers. However, *Fusarium* species composition differed between different soil layers due to different survival structures of the species [31]. Especially *F. verticillioides* survived best in maize stalks at 30 cm depth due to higher moisture content and poor decomposition of plant tissue [60]. According to this study and previous research, weather conditions had the largest influence on the local *Fusarium* species composition and disease incidence in maize, however, prevention and management practices including crop rotation and tillage types may also affect ear and stalk rot diseases and mycotoxin accumulation [2,9,12,66,67].

In the three years of investigation (2016–2018), *F. graminearum*, *F. verticillioides* and *F. temperatum* were the most frequent *Fusarium* species on maize in Germany. A high year-to-year variability was observed in the shift of species composition towards a high occurrence of *F. verticillioides*. Increasing temperatures and dry periods in summer can affect the *Fusarium* species complex and increase the risk of contamination with fumonisin-producing species such as *F. verticillioides* and *F. temperatum* [48]. In addition, feeding of the European corn borer (*Ostrinia nubilalis*) and the Western corn rootworm (*Diabrotica virgifera*) in Germany will likely further enhance disease incidence and mycotoxin contamination of ears and stalks as well as root rots in maize [11,16,68]. The current expansion of maize acreage and shorter crop rotations with other small grain cereals due to renewable energy policy will further increase the risk of *Fusarium* infection and mycotoxin contamination.

The current results emphasize the importance of further studies of the impact of changing climatic conditions and its interplay with cultural practices on the development of *Fusarium* population and the mycotoxin contamination of maize crops.

## 4. Materials and Methods

### 4.1. Sampling and Isolation

Naturally *Fusarium*-infected maize ear and stalk samples were collected from silage and grain maize fields in Germany in 2016 (94 ears from 18 locations), 2017 (180 ears from 42 locations and 110 stalks from 21 locations) and 2018 (113 ears from 18 locations and 80 stalks from 14 locations). For each sample site agronomic data like soil tillage and previous crop as well as meteorological data such as precipitation, air temperature, and humidity during the vegetation period were recorded. Five to nine *Fusarium*-infected ears or stalks per location were placed in paper bags and sent to the Plant Pathology and Plant Protection Division in Göttingen, Germany for further analysis. Disease severity on kernels and rachis was scored according to EPPO Guidelines [69].

Thirty randomly chosen kernels of each ears were surface sterilized for 10 min with 0.1% silver nitrate and incubated on moist sterile filter paper for two days at room temperature. Afterwards, kernels with outgrowing *Fusarium* mycelium were placed on potato dextrose agar (PDA). The rachis was cut in nine slices, three from the base, three from the middle part and three from the tip of the ears. The slices were surface sterilized as described above and placed directly on PDA plates. The stalk samples were cut in nine slices, three from the lower nodium, three from the internodium and three from the upper nodium. The samples were surface sterilized and placed on PDA plates as the rachis slices. After two days, presumed *Fusarium* mycelium outgrown from the samples was transferred to synthetic low nutrition agar (SNA) to produce single spore cultures. The isolates were stored as single spore cultures on synthetic SNA plates at 4 °C.

The ear and stalk infection were calculated by the following equation:

(1) $$\mathrm{Ear}\;\mathrm{infection}\;\left[\%\right]=\frac{\begin{array}{c} \mathrm{Number}\;\mathrm{of}\;\mathrm{Fusarium}\\ \;\mathrm{infected}\;\mathrm{kernels}\;\mathrm{per}\;\mathrm{cob} \end{array}}{\begin{array}{c} \;\mathrm{Number}\;\mathrm{of}\;\mathrm{kernels}\;\\ \left(n=30\right) \end{array}}\;÷\;\mathrm{Cobs}\;\mathrm{per}\;\mathrm{location}$$

(2) $$\mathrm{Stalk}\;\mathrm{infection}\;\left[\%\right]=\frac{\begin{array}{c} \mathrm{Number}\;\mathrm{of}\;\mathrm{Fusarium}\;\\ \mathrm{infected}\;\mathrm{slices}\;\mathrm{per}\;\mathrm{stalk} \end{array}}{\begin{array}{c} \mathrm{Number}\;\mathrm{of}\;\mathrm{slices}\;\\ \left(n=9\right) \end{array}}\;÷\;\mathrm{Stalks}\;\mathrm{per}\;\mathrm{location}$$

### 4.2. Species Identification

In vitro cultures of *Fusarium* were identified macroscopically by colony characters on PDA and microscopically on SNA [70]. Total DNA was extracted from lyophilized mycelium from single spore cultures by using a CTAB-based protocol as described previously [71]. Standards of genomic DNA were obtained from *F. temperatum* MUCL52463 and *F. subglutinans* CBS215.76 [45]. The quality and quantity of extracted DNA were assessed after electrophoretic separation in agarose gels (0.8% (w/v) stained with ethidium bromide. The electrophoresis was carried out for 60 min at 4.6 V/cm.

Partial translation elongation factor 1-alfa (*tef1α*) nucleotide sequence was used to differentiate between *F. temperatum* and *F. subglutinans*. Amplification was performed in a peqSTAR96 thermocycler (PEQLAB, Erlangen, Germany) using 1:100 dilutions of DNA extracts in a total reaction volume of 25 µL.

The *tef1α* gene was amplified using primers EF1 (ATGGGTAAGGARGACAAGAC) and EF2 (GGARGTACCAGTSATCATGTT) [72] in a PCR reaction consisting of *Taq* reaction buffer (10 mM Tris-HCl, 50 mM KCl, 1.5 mM MgCl_2_, pH 8.3 at 25 °C), 100 µM of each deoxyribonucleoside triphosphate, 0.3 µM of each primer, 0.62 U HotStart-polymerase (NEB) and 1 µL template DNA solution. The final MgCl_2_ concentration was adjusted to 2 mM. The PCR cycling conditions for the amplification of *tef1α* included an initial denaturation for 30 s at 95 °C; 30 cycles consisting of 30 s at 94 °C, 30 s at 58 °C, and 1 min at 68 °C; and final extension for 5 min at 68 °C. *Fusarium* species were identified by multiple alignment of each sequence with the sequences of standard strains and other reference sequences using ClustalW [73] in MEGA version 7.0.2 [74].

### 4.3. Meteorological and Agronomical Data

The meteorological data were obtained from the closest weather stations (<10 km) to the sample location. In Bavaria, meteorological data were received from AgrarMeterologie of the Bavarian state research center for agriculture (https://www.wetter-by.de). The air temperature and the relative air humidity were recorded as daily means and precipitation as monthly sum from May to September. The agronomical field data of tillage and previous crop were obtained from breeding companies and farmers. The previous crop was assigned to four categories, in maize (silage maize and grain maize), wheat (winter wheat), sugar beet and other crops (potato, cabbage, strawberry, rye, barley). Soil tillage was differentiated into two groups; ploughing (moldboard ploughing) and no ploughing or reduced tillage including chisel ploughing and rotary harrow.

### 4.4. Statistical Analyses

Statistical analyses were performed using Statistica version 13.3 (TIBCO^®^ Data Science, Palo Alto, CA, USA). Non-parametric data of average infection, tillage treatments and previous crop were statistically analyzed using Mann–Whitney-U-Test and Kruskal–Wallis-ANOVA. Tests were performed at a probability level of 95%. Pearson’s correlation coefficients were used to examine the relationship between temperature and precipitation in June, July, August, and September and infection with predominant *Fusarium* species occurring on ear and stalk samples. In addition, a multiple regression was performed to determine the relationship of ear and stalk infection with temperature and/ precipitation in July for each sample location. The impact of weather conditions, soil tillage or pre-crops on the occurrence of *Fusarium* species was analyzed by variance components derived from the overall variance estimated with the restricted maximum likelihood model.

## Figures and Tables

**Table 1:** Percentage of ears and stalks infected with *Fusarium* species.

	Ears Infection							Stalk Infection					
*Fusarium* Species	Frequency [%]				Sample Sites			*Fusarium* Species	Frequency [%]			Sample Sites	
	2016	2017	2018	Total	2016	2017	2018		2017	2018	Total	2017	2018
	n = 94	n = 180	n = 113		n = 18	n = 42	n = 18		n = 110	n = 80		n = 21	n = 14
*F. gramineaum*	79	71	30	60	17	41	15	*F. graminearum*	81	43	62	20	11
*F. verticillioides*	19 ^2^	13	39	24	11	11	16	*F. equiseti*	11	34	22	10	9
*F. temperatum*	33 ^1^	15	21	23	11	21	15	*F. culmorum*	22	16	19	14	11
*F. poae*	11	15	12	14	6	11	12	*F. temperatum*	15	20	17	7	13
*F. cerealis*	11	12	3	9	6	13	2	*F. cerealis*	19	15	17	9	10
*F. proliferatum*	**	4	13	6	**	3	12	*F. verticillioides*	7	9	8	6	3
*F. tricinctum*	4	7	2	5	3	8	3	*F. avenaceum*	6	5	5	5	3
*F. avenaceum*	10	5	1	5	4	8	1	*F. tricinctum*	5	8	6	4	5
*F. culmorum*	1	5	4	4	1	9	3	*F. proliferatum*	3	11	6	3	5
*F. subglutinans*	*	2	2	2	*	3	3	*F. poae*	3	5	4	3	3
								*F. subglutinans*	1	3	2	1	2
*F. sporotrichoides*	4	1	5	2	3	1	4	*F. sporotrichoides*	1	0	1	1	0

^1^ In 2016, there was no differentiation between *F. subglutinans* and *F. temperatum.* ^2^ In 2016, there was no differentiation between *F. verticillioides* and *F. proliferatum.*

## Supplementary Materials

The following are available online at https://www.mdpi.com/2076-0817/9/3/236/s1, Table S1: Mean monthly air temperature [°C] (AT), mean relative humidity [%] (RH) and cumulative monthly precipitation [mm] (PP) in June, July, August and September in the year 2016, 2017 and 2018 within the sampling locations.
