# Supplementary material for: Impact of Environmental Conditions and Agronomic Practices on the Prevalence of Fusarium Species Associated with Ear- and Stalk Rot in Maize
Source: Pathogens. 2020 Mar 21;9(3):236. doi: 10.3390/pathogens9030236 (PMC7157686; doi:10.3390/pathogens9030236)
Supplement: Supplementary file 1 [file pathogens-09-00236-s001.pdf]

Table S1. Mean monthly air temperature [°C] (AT), mean relative humidity [%] (RH) and cumulative monthly precipitation [mm] (PP) in June, July, August and September in the year 2016, 2017 and 2018 within the sampling locations.

2016	June			July			August			September		
Location	AT [°C]	RH [%]	PP [mm]	AT [°C]	RH [%]	PP [mm]	AT [°C]	RH [%]	PP [mm]	AT [°C]	RH [%]	PP [mm]
Altötting	16.8	86	132	19.1	74.3	153.6	17.6	85.8	65.1	15.9	90.1	149.6
Braunau am Inn	18	77	163	19.9	75.1	140	18.9	75.3	92.5	17.5	77.3	103.6
Bad Lauchstädt				17.8	71.2	52.2						
Einbeck				18.57	75.71	65.2						
Frankendorf				18.9	77	70.6						
Fraunberg-Grucking				18.9	77	71						
Grucking	16.5	79	104	18.8	77.2	70.6	17.7	75.6	61.1	15.5	79.8	58.8
München/Bockhorn	16.8	78	98.4	19.1	74.8	88.2	17.8	76.8	101	15.7	80.9	70.8
Löningen				18.5	76	33						
Osterhofen	17.5	80	123	19.0	77.35	80.4	18.4	78.3	47	16.9	80.3	82.5
Ostbevern				18.8	77	78						
Reith				19.5	76.4	129.3						
Pocking 1	17.9	85	194	19.7	84.5	168	18.2	86.9	76	16.1	89.1	121.7
Pocking 2	17.9	85	194	17.9	76.5	129.3	18.2	86.9	76	16.1	89.1	121.7
Rustenhard				16.4	78.7	87.2						
Tönisvorst				19.5		35.9						
Thenn	16.5	79	104	19.1	74.8	88.2	17.7	75.6	61.1	15.5	79.8	58.8
Unterneukirchen				19.4	74	153						
Wadersloh	17.4	78	130	19.31	71.7	43	18.6	70.4	55	18.9	64.4	16.2
Weihmörting	17.9	85	194	19.6	76	129	18.2	86.9	76	16.1	89.1	121.7
Wesel				19.2		62.8						
**MEAN**	17.3	80.8	138.1	18.9	76.1	91.8	18.1	79.1	70.5	16.5	81.2	87.1

2017	June			July			August			September		
Locations	AT [°C]	RH [%]	PP [mm]	AT [°C]	RH [%]	PP [mm]	AT [°C]	RH [%]	PP [mm]	AT [°C]	RH [%]	PP [mm]
Altötting	20	66	59	19.2	82.2	166.4	20	74	84	13	83	31
Bad Lauchstädt	18	68		19	73		19	72		14	80	
Bernburg	19.1	63.4	67.5	19.2	70.5	89.7	19.2	70.2	99.8	14.7	73.7	39.7
Borken	18	69.8	46.8	18	78.1	121.2	22.9	81.4	55.7	18.9	82.6	115.1
Cloppenburg	17.5	70.6	93.2	17.5	77.0	150.4	17.4	75.9	58.8	13.7	82.3	85
Dinkelsbühl	18	67.3	119.9	18	73.4	134.5	19	75.1	77.4	12	81.7	71
Einbeck				18.2	80.2	174						
Geldern	18.9	69.8	68.5	18.9	75.8	109.7	18.1	79.7	67	14.3	85.3	85.4
Giebelstadt	18.5	67.2	70.5	18.9	72.2	105.9	18.7	74.0	71.5	13.0	78.1	74.6
Gondelsheim				20.5	74.1	109.3						
Greven				18.3	79							
Grucking	19	68		18.7	74.5	140	19	76		12	81	
Haselünne	18	69		17.8	83.4	89.2	17	77		13	84	
Heilbronn	20	64		20.1	73.7	114.2	19	76		13	82	
Herzlake				17.8	83.49	89.2	17	77		13	84	
Ichenhausen				18.2	82.4	113.3						
Kleinwanzleben				19.1		98.6						
Löningen	17.7	73.1	73.1	18	78.3	100.6	17.4	78.9	65.6	13.4	88.4	83.9
Moosham	19	64.4	56.1	19	70.9	80.4	19.2	74.6	103.2	12.1	84.1	41.1
Möttingen	18.9	66.2	62.8	19.1	71.1	99.1	19.0	75.5	116	12.8	78.9	72.3

Münzesheim	23	54		20.5	74.6	109.3	22	66		16	73	
Neumarkt (St.Veit)		70.2	65.7	18.8	72.3	96.6		78.3	91.5		84.1	56.9
Neupotz				20.7	75.8	61.8						
Neuhaus am Inn		64	47.6	19	72.4	93.3	19.2	75.1	112.9	12.3	85.2	55.9
Ostbevern				18.8		119.7	18	77		14	84	
Osterhofen		67		18.9	76	93.5	19	75		12	82	
Plessa		64.9	64.7	19.5	70.9	90.7		70.9	65.9		78	31.1
Pocking	21	58		19.5	81.8	94.3	21	67	126	13	77	32
Pritzwalk		77.3	113.5	17.1	79.8	84.6		77.6	29.9		83.4	54.8
Reith				19.5	72.8	101.2			81			60
Rheine	18.3	66.3	71	18.2	74.7	142.9	17.7	75.1	71.5	13.8	81.9	93.3
Saerbeck												
Sankt Peter am Hart				19.5	82.8	121.6						
Soest-Epsingen				18.5	74.7	109						
Tönisvorst				18.1								
Ulm			75	18.3	82.4	113.4			92			56
Wadersloh-Liesborn	18.6	66.8	55.7	18.6	74.4	144.4	18.0	76.9	94.2	13.9	81.1	108
Welbhausen			69	18.9	75.4	112.2			53.1			60.4
Wesel				18	82.4	90.1						
**MEAN**	**19.0**	**66.8**	**71.1**	**18.8**	**76.5**	**110.4**	**19.0**	**75.1**	**80.9**	**13.6**	**81.6**	**65.4**

**2018**	**June**			**July**			**August**			**September**		
**Location**	**AT [°C]**	**RH [%]**	**PP [mm]**	**AT [°C]**	**RH [%]**	**PP [mm]**	**AT [°C]**	**RH [%]**	**PP [mm]**	**AT [°C]**	**RH [%]**	**PP [mm]**
Altötting	17.9		118.9	19.9	71	68.5	19.2		146.7	14.9		76.7
Braunau	18.3		63.2	21.1	-	79	20.4		86.4	15.2		55.7
Bernburg												
Einbeck				22.6	63.4	56.2						
Gondelsheim	19.4	75.1	60.8	22	64.6	46.9	21.7	66.8	38.3	16.6	71.6	23.9
Grucking	17.7	78.6	123	19.3	76.65	57.9	19.8	78.3	66.7	14.7	85.2	56.9
Hohenheim				21	-	-						
Kleinwanzleben				22.2	-	29.4						
Künzing	18.2	75.2	68.3	19.3	71.0	30.9	20.4	70.2	38.7	14.8	81.0	64.2
Löningen				20.3	-	14.4						
Mariaposching	18.9	71.5	90.5	20.4	67.7	24.3	21.3	67.1	46	15.5	80.5	71.7
Mintraching	18.9	69.2	80.2	20.5	66.9	38.6	21.2	67.2	43.4	15.2	79.8	52.7
Neuhaus am Inn	18.9	77.4	85.2	20.0	77.1	35.3	21.0	77.7	87.4	15.3	85.7	79.2
Ostbevern				21.5	-	8.9						
Osterhofen	19.6	66.9	37.7	19.2	76	74.8	19.1	77.3	79.7	12.5	86.2	30.4
Pocking	18.9	77.4	85.2	20	77.1	35.3	21	77.4	87.4	15.3	85.7	79.2
Prenzlau	18.0		43.1	20.4		61.9	20.8		17.3	16		11.3
Reith	16.5	85.1	124.2	18.9	79.2	69.8	19.6	80.0	111.8	14.7	88.5	112.4
St. Andreas-berg				-	-	-						
Tönisvorst				22.2	-	5.8						
Triftern	18.3	76.2	63.2	19.5	75.8	79	20.4	77.6	86.4	15.2	86.8	55.7
Ulm	17.4	83.1	47.1	19	79.3	40.9	19.4	79.2	46	14.9	83.4	29.8
Wesel												
**MEAN**	**18.3**	**75.8**	**81.7**	**20.6**	**72.1**	**40.4**	**20.3**	**73.7**	**71.9**	**14.9**	**82.4**	**59.4**
